# A Focus on Intra-Abdominal Sepsis with Biomarkers: A Literature Review

**DOI:** 10.5152/eurasianjmed.2022.22296

**Published:** 2022-12-01

**Authors:** Rıfat Peksöz, Enes Ağırman, Fuat Şentürk, Yavuz Albayrak, Sabri Selçuk Atamanalp

**Affiliations:** Department of General Surgery, Atatürk University Faculty of Medicine, Erzurum, Turkey

**Keywords:** Intra-abdominal infection, biomarkers, inflammatory mediators, sepsis-3

## Abstract

Sepsis is defined as life-threatening organ dysfunction caused by a dysregulated host response to infection according to the Third International Consensus Definitions for Sepsis and Septic Shock definitions. It is a clinical condition with high morbidity and mortality due to its complex pathophysiology and lack of a complete treatment. It constitutes a significant economic burden because it constitutes a substantial part of intensive care patients, and the treatment process is lengthy and costly. Therefore, early diagnosis and treatment of the disease are essential.

After pneumonia, an essential source of sepsis is intra-abdominal infection. Due to the presence of multiple and polymicrobial sources of infection, abdominal sepsis progresses more seriously. The effective treatment of intra-abdominal infection consists of early recognition of the disease, control of the source, appropriate antibiotic therapy, and stabilization in the intensive care setting with an excellent surgical approach.

We searched PubMed, EMBASE, MEDLINE, and the Cochrane Library. Two authors reviewed all identified abstracts and selected articles for full-text review. We included original studies assessing mediators in intra-abdominal sepsis.

Inflammatory and protein mediators such as acute phase protein and chemokine cytokines play an essential role in intra-abdominal sepsis. In clinical practice, white blood cell count, C-reactive protein, and procalcitonin are the most used parameters in the definition of abdominal infection. Tumor necrosis factor-alpha, interleukin-6, high-mobility group protein B1, and presepsin are other markers with high diagnostic efficiency, even though they are not used routinely. Despite everything, there is a need for highly effective markers that can be used in the diagnosis and follow-up of sepsis. Great hope is attached to these markers.

This review aims to discuss the importance of the most used markers in the diagnosis and follow-up of abdominal sepsis and the markers on which there are essential studies in light of current literature.

**Main Points**

- Sepsis is defined as life-threatening organ dysfunction caused by a dysregulated host response to infection according to sepsis-3 definitions.
- One of the essential sources of sepsis is abdominal infections.
- Intra-abdominal sepsis has high morbidity and mortality due to multiple and polymicrobial infection sources.
- In clinical practice, white blood cell count, C-reactive protein (CRP), and procalcitonin (PCT) are the most used parameters in the definition of abdominal infection.
- Persistent elevation of CRP or PCT levels in blood appears to be correlated with septic complications.

## Introduction

At the present time, the rate of septic diseases is increasing rapidly due to the increasing elderly population. Considering the patients with suspected sepsis but not registered, the actual rate is higher than reported in the literature. Despite advances in all diagnostic and treatment modalities, sepsis remains the leading cause of death in surgical intensive care units. It creates a severe economic burden due to the complex and long-term treatment. Therefore, early diagnosis and treatment of the disease are essential.^1-4^

According to previous definitions, sepsis has been defined as a systemic inflammatory response syndrome (SIRS) against infection. It was called severe sepsis when organ failure was added and septic shock when cardiocirculatory failure was added to SIRS.^5^ However, due to the deficiencies in these definitions, the sepsis criteria were revised in the Third International Consensus Definitions for Sepsis and Septic Shock (Sepsis-3) in February 2016. The use of the SIRS criteria and the definition of severe sepsis have been abandoned in this consensus. Sepsis is defined as life-threatening organ dysfunction caused by a dysregulated host response to infection according to sepsis-3 definitions. According to this classification, a Sequential Organ Failure Assessment (SOFA) score of ≥2 points due to intra-abdominal infection (IAI) was considered abdominal sepsis.^6^ In determining the bedside sepsis-related prognosis in adult patients with suspected disease in the out-of-hospital, emergency room, or general hospital conditions, quick SOFA can be used. Quick SOFA uses these 3 criteria: hypotension, altered mental status, and tachypnea.^7^

Septic shock is the presence of sepsis with (despite adequate volume resuscitation) the persistent hypotension-requiring vasopressor drugs to maintain mean arterial pressure ≥ 65 mm Hg and lactate level ≥ 2 mmol/L. Mortality in these patients is over 40%. Hypoperfusion in sepsis impairs tissue perfusion and microcirculation. Changes in metabolism cause lactic acidosis, oliguria, and changes in mental status. These changes herald the development of multiple organ dysfunction syndromes.^6,7^

Intra-abdominal infection is the second most common cause of sepsis after pulmonary focus.^8^ Intra-abdominal infections are separated into 2 parts, uncomplicated or complicated, according to the width of the infection. An uncomplicated IAI rarely causes a critical illness with organ failure. On the other hand, complicated IAI caused by disruption of the gastrointestinal tract or other hollow organs causes localized or diffuse peritonitis. This causes organ failure and, ultimately, abdominal sepsis. Abdominal sepsis may occur due to spontaneous perforation (e.g., gastric ulcer perforation), complicated diverticulitis, or complication of elective abdominal surgery. Intestinal perforation and other surgical complications arise after surgery, especially in a significant portion of the patients in the surgical intensive care unit.^3,9,10^ Complicated IAI represents the second most common cause of infectious morbidity and mortality after pneumonia.^11^ Sixty-six percent of surgical sepsis patients are due to IAIs. The mortality of abdominal sepsis varies between 7.6% and 36%. Again, the majority of surgical patients occur after elective surgery.^1-3^

The increase in the number of IAI patients continues. The disease becomes more fatal due to multiple and polymicrobial sources of infection.^12^ Effective treatment of IAI consists of early recognition of the disease, control of the source, appropriate antibiotic treatment, and stabilization in the intensive care setting with a good surgical approach.^13^ To better understand the pathogenesis and management of intra-abdominal sepsis, many experimental studies have been conducted and continue to be conducted on polymicrobial sepsis generation and preventive treatment.^14-18^

Sepsis is the interaction between the host and pathogens. In this effect, biochemical mediators are produced, and inflammatory cascades are triggered.^19^ The pathogenesis of sepsis involves the activation and release of stress hormones, hundreds of mediator molecules, cytokines, and acute phase proteins, which can be considered prognostic biomarkers.^20^

Early diagnosis and timely treatment will improve the results of the disease. Thus, a method that can diagnose sepsis in the early phase, make an early prognostic evaluation, and fully determine the effectiveness of treatment and surgical management gains vital importance. Great hope has been placed on biomarkers as a method to cope with these problems.^11^

This review article aims to investigate the significance of inflammatory mediators for abdominal sepsis. Again, we discussed the value of these mediators in the early diagnosis of intra-abdominal sepsis and their significance in not predicting the outcome of the disease in light of current literature.

## Methods

We searched PubMed, EMBASE, MEDLINE, and the Cochrane Library database, limited to English. Two authors (R.P., Y.A.) reviewed all identified abstracts and selected articles for full-text review. We included all articles that were reviews, meta-analyses, and original studies (cohort study, experimental study) assessing protein or inflammatory mediators about intra-abdominal sepsis. Articles that were letters, conference abstracts (no full-text available), and written articles about pediatric patients were excluded. Keywords for searching included abdominal sepsis, biomarkers, and inflammatory mediators.

### Biomarkers

The metabolic, hemodynamic, and immune changes seen in sepsis occur through cytokines and mediators that play a role in intercellular signal transmission.^4,21^ These biomarkers include measurement of acute-phase proteins, endothelial cell markers, chemokines, cytokines, leukocyte surface markers, damage-associated molecular patterns (DAMPs), non-coding ribonucleic acids (RNAs), micro RNA, and soluble receptors, as well as metabolites and alterations in gene expression (transcriptomics).^22^

Biomarkers are measurable indicators used to define whether the disease state is normal or pathogenic. Biomarkers are markers that can be used to diagnose and treat disease, especially surgical diseases such as acute abdomen, cancer, etc. One of the most used methods in diagnosing and following many diseases is biomarkers.^23-25^ These markers can be used alone or with other auxiliary methods. Biomarkers play a critical role in the diagnosis of the disease, early recognition of organ dysfunction, disease risk classification, prognosis, and patient management.^22^

Many markers have been identified as early markers of post-surgical infectious complications. In this study, especially the most commonly used markers with high diagnostic efficiency were discussed. It has been mentioned in some recent and experimental studies on new markers.

## History of Mediators

C-reactive protein (CRP) is the oldest and most widely used mediator. Later, interleukins (IL-6, -8, and -10) and tumor necrosis factor-alpha (TNF-α) were reported as the most used markers. After 2000, procalcitonin (PCT) became a new marker in studies. More recently, endothelial dysfunction molecules and DAMPs have been added to the ever-growing list of inflammatory mediators.^19^

In clinical practice, white blood cell (WBC) count, CRP, and PCT are the most used parameters in the definition of abdominal infection.^11,26,27^

## Role of White Cell Count in the Diagnosis of Sepsis

White blood cell is one of the most widely used and easy-to-work hematological markers, especially in the diagnosis of inflammatory diseases. It is a marker with high diagnostic efficiency, especially in surgical conditions such as acute appendicitis.^28,29^ The diagnostic value of WBC in sepsis is limited. However, the criteria in the initial SIRS definition included an increase or decrease in the WBC count (>12 000 or <4000/mm^3^) or a normal WBC count with >10% bands. White blood cell levels can be raised in inflammation with non-infectious causes, making it rarely helpful as a sole diagnostic marker.^5,30^ However, one of the WBC components, neutrophil, may be more valuable for sepsis. Neutrophil count increases during inflammation and relatively leads to lymphopenia. Thus, the neutrophil-to-lymphocyte ratio (NLR) increases. Neutrophil-to-lymphocyte has been used for the early prediction of inflammation in cases of mesenteric ischemia, acute appendicitis, and incarcerated hernia.^24,31,32^ Again, in intra-abdominal sepsis studies, the NLR value was associated with sepsis and its severity and was shown to be more significant than the WBC value.^33,34^

## C-Reactive Protein

The CRP and PCT are the most used markers to diagnose or exclude the diagnosis of IAI and have high diagnostic efficiency.^13,35^ Postoperative serum CRP and PCT levels increase on the first postoperative days 2 and 3. It reaches the peak level in the day and goes down to the normal level on the post-op seventh day. Postoperative increasing CRP levels after the fifth day suggest septic complications, such as anastomotic leakage or abscess.^19^

## Procalcitonin

Procalcitonin is primarily secreted by the parafollicular C-cells of the thyroid gland and is the precursor of calcitonin. In the case of bacterial infection, cytokines, lipopolysaccharides (LPS), neutrophils, and brain, intestine, lung, and liver cells stimulate PCT production.^36^ The PCT level is increased in bacterial and fungal infections but not in viral and non-infectious inflammation conditions. Procalcitonin is used in the diagnosis of IAIs and to guide antibiotic therapy.^13^ In the diagnosis of postoperative sepsis, ever-increasing PCT levels provide more valuable information in the diagnosis of postoperative sepsis than other markers. A PCT level of >1.1 ng/mL on the postoperative first day after major surgery is a precursor to complications such as anastomotic leakage or pneumonia, or sepsis to occur.^37^

## Interleukin-6

Interleukin-6 is one of the most used inflammatory indicators to evaluate infectious comorbidities. It is released in the early phases of inflammation. Interleukin-6 can also regulate hematopoietic function, immune response, and acute-phase response. Since it increases in correlation with the severity and duration of the infection, this marker is a valuable marker for predicting the prognosis and outcome of patients with sepsis.^38^ In a recent study, Song et al^38^ found that serum expression of TNF-α and IL-6 were associated with IAIs in patients after surgery. A composite of IL-6, PCT, and TNF-α can also increase the reliability of the prediction of postoperative infections. In coronavirus disease-2019 patients, which is also a current disease, IL-6 is an early prognostic inflammatory mediator that determines the severity of the disease and IL-6 inhibitors can be used in the treatment of the disease.^39,40^

## Tumor Necrosis Factor-Alpha

Tumor necrosis factor-α is the major pro-inflammatory cytokine that plays a vital role in the anti-inflammatory and antimicrobial response by activating apoptosis of lymphocytes, cell proliferation, differentiation, and leukocytes.^41^ It is the earliest released and the most potent pro-inflammatory cytokine. Its release is stimulated in cases of injury and bacterial infection. Tumor necrosis factor-α peaks 6 hours after induction of sepsis and significantly increases in septic shock, correlated with the severity of the disease. Tumor necrosis factor-α ≥ 39.4 can be used as a predictor of sepsis risk.^20,41^ High concentrations of TNF-α can be used to predict septic infection risk. It plays a central role in TNF-α systemic inflammatory response. It directly affects septic shock, and the levels of this marker are associated with sepsis-induced death.^41^

Serum assays of several other markers, such as soluble CD14 subtypes (presepsin), proadrenomedullin, cytokines, DAMPs, or endothelial dysfunction molecules, have been proposed but are not commercially available for routine follow-up. Extensive cohort studies of these mediators are needed in the diagnosis of IAI.^19^

## Damage-Associated Molecular Patterns

Tissue damage and shock lead to an extracellular release of associated molecular patterns (DAMPs). This leads to SIRS and hypoxia, reducing the resistance to infection and increasing the risk of sepsis.^42^ The best-known DAMPs is high-mobility group B1 (HMGB1). High-mobility group B1 is a deoxyribonucleic transcription factor that acts as a pro-inflammatory cytokine secreted from innate immune cells such as neutrophils, monocytes, and macrophages. In acute appendicitis studies, it gave more significant results than the WBC level.^43^ There is a positive correlation between the severity and morbidity of the disease and the HMGB1 level in surgical patients. In another study, HMGB1 level was found to be higher in peritonitis than in control patients.^44^ Compared with TNF-α and IL-1, it increases in the late phases of sepsis.^4^ Albayrak et al^45^ found that inhibiting HMGB1 may reduce inflammation, fibrosis, and apoptosis and stop the progression of chronic liver disease.

## Presepsin (Soluble CD14 Subtype)

In the last 10 years, many studies have been conducted on the effect of presepsin on sepsis, and it has been shown as a marker with high diagnostic value. Presepsin is a LPS-binding protein (LPS–LBP) complex receptor and is thought to be a fragment of CD14. Presepsin (soluble CD14 subtype) is generated in the bacterial infection condition and is expressed explicitly in sepsis.^26^ Presepsin (soluble CD14 subtype) has the highest diagnostic accuracy for sepsis. Increased serum presepsin level correlates with PCT levels and reflects poor outcomes in patients with sepsis.^26,46^ In a recent study, the diagnostic efficiency of presepsin was higher than CRP and PCT in confirming the definition of abdominal sepsis.^47^

## Intraperitoneal Mediators

In patients with peritonitis, the peritoneal level of inflammatory mediators is 10-1000 times higher than the blood level. Measurement of peritoneal cytokine may be more valuable for assessing and monitoring the patient's reaction to inflammation.^19^

## Other Less-Used Current Markers and New Therapeutic Agents or Novel Drug Targets

Some markers have not been applied in routine clinical practice but have recently been studied for their effectiveness. They can be used in the future diagnosis and follow-up of intra-abdominal sepsis. Markers, such as neutrophil CD64,^11^ pancreatic stone protein,^48^ 5-hydroxyindoleacetic acid,^49^ and Trefoil factor 3,^50^ will better demonstrate their value in future studies. Since sepsis's pathogenesis is complex and accepted as a systemic disease, it is difficult to treat, and a standard treatment modality has not been determined yet.

## Conclusions

Intra-abdominal infection is one of the leading causes of sepsis. The treatment process is complicated, and mortality is high. Although serum PCT and CRP are the most valuable markers in the diagnosis and treatment process of the disease, an ideal marker has not been determined yet. The issue of sepsis will continue to be up-to-date due to its economic consequences and the fact that it is still a disease with a very high mortality rate. Randomized controlled studies on this subject with large patient groups are needed to identify the ideal biomarkers and explain the roles of mediators in intra-abdominal sepsis. However, finding ideal markers will be difficult due to the complexity of sepsis and pathophysiological mechanisms.
